# Discovery of an APP-selective BACE1 inhibitor for Alzheimer's disease

**DOI:** 10.1016/j.neurot.2025.e00610

**Published:** 2025-05-20

**Authors:** Jesus Campagna, Barbara Jagodzinska, Dongwook Wi, Chunni Zhu, Jessica Lee, Whitaker Cohn, Michael Jun, Chris Elias, Samar Padder, Olivier Descamps, Clare Peters-Libeu, Qiang Zhang, Olivia Gorostiza, Karen Poksay, Patricia Spilman, Dale Bredesen, Varghese John

**Affiliations:** a Drug Discovery Lab (DDL), Department of Neurology, Easton Center for Alzheimer's Disease Research and Care, David Geffen School of Medicine, University of California, Los Angeles (UCLA), CA, 90095, USA; b Buck Institute for Research on Aging, Novato, CA, 94945, USA

**Keywords:** ASBI, BACE1 inhibitor, APP-selective, FAH65

## Abstract

Inhibition of amyloid precursor protein (APP) beta-site cleaving enzyme 1 (BACE1) has been a target for Alzheimer's disease (AD) therapeutic development. Here, we report our identification of APP-selective BACE1 (ASBI) inhibitors that are selective for APP as the substrate and BACE1 as the target enzyme. A known fluoro aminohydantoin (FAH) inhibitor compound was identified by screening a compound library for inhibition of BACE1 cleavage of a maltose binding protein (MBP)-conjugated-APPC125 substrate followed by optimization and IC50 determination using the P5-P5′ activity assay. Optimization of the screening hit led to candidate FAH65, which displays selectivity for inhibition of APP cleavage with little activity against other BACE1 substrates neuregulin 1 (NRG1) or p-selectin glycoprotein ligand-1 (PSGL1). FAH65 shows little inhibitory activity against other aspartyl proteases cathepsin D (Cat D) and BACE2. FAH65 reduces BACE1 cleavage products soluble APPβ (sAPPβ) and the β C-terminal fragment (βCTF), as well as amyloid-β (Aβ) 1-40 and 1-42, both *in vitro* in cells and *in vivo* in an animal model of AD. In a murine model of AD, FAH65 improved the discrimination score in the Novel Object Recognition (NOR) memory testing paradigm. The active enantiomer of racemate FAH65, FAH65E(-), displays good brain-penetrance and target engagement, meriting further pre-clinical development as an ASBI that may reduce Aβ levels and overcome the deleterious effects of the non-selective BACE1 inhibitors that have failed in the clinic. FAH65E(-) has the potential to be a first-in-class oral therapy that could be used in conjunction with an approved anti-Aβ antibody therapy for AD.

## Introduction

Brain tissue of Alzheimer's disease (AD) patients is characterized by the presence of neuritic plaques largely composed of amyloid-β (A*β*) [1,2] and neurofibrillary tangles resulting from the hyperphosphorylation of the protein tau [[3], [4], [5]]. A*β* is the product of step-wise cleavage of amyloid precursor protein (APP) by the *β*-site APP-cleaving enzyme 1 (BACE1) and *γ*-secretase [6,7]. In an alternative cleavage pathway, α-secretase (putatively ADAM10) cleavage of APP results in production of trophic, synapse-supporting peptides soluble APPα (sAPPα) and the α C-terminal fragment (αCTF) [[8], [9], [10]].

While the amyloid cascade hypothesis of AD has come under scrutiny in the last decade due to the failure of many Aβ- and BACE1-directed potential therapies in the clinic [11], it is supported by the genetics underlying familial forms of the disease that enhance amyloid production [12,13], by identification of gene variants that confer protection against cognitive decline [14]; and by recent clinical findings that the amyloid-directed antibodies aducanumab (Aduhelm®), lecanemab (Lequembi®), and donanemab (Kisunla®) slow cognitive decline in early AD [[15], [16], [17], [18]].

Antibody-based biologic therapies, despite effectively clearing Aβ deposits in the brain as seen by PET imaging, present challenges as feasible therapeutics due to their limited brain-permeability, high cost, and requirement for IV infusion at hospitals or infusion centers [19]. An orally-available, brain-permeable small-molecule therapeutic that inhibits BACE1 cleavage of APP would overcome those limitations and, as a maintenance therapy, would not only decrease amyloid plaque formation, but also the upstream formation of the Aβ oligomers that are implicated in synaptic loss and cognitive decline [11,20].

The discovery that an A673T (Icelandic) mutation in APP at the P2′ residue of the BACE1 cleavage site reduces BACE1 cleavage of APP and protects against both AD and other age-related cognitive decline [14] provides further strong support for the hypothesis that Aβ production - and specifically BACE1 cleavage - is a critical event in AD pathogenesis. Thus, inhibition of BACE1 as a strategy for therapeutic development for AD has been a focus of many research groups.

A critical limitation of BACE1 inhibitory strategies is the potential inhibition of cleavage of non-APP substrates, resulting in side effects. Inhibition of non-APP substrates such as neuregulin 1 (NRG1), p-selectin glycoprotein ligand 1 (PSGL1) and gp-130 raise concern [[21], [22], [23], [24], [25]]. Therefore, the optimal BACE1 inhibitor would be one that would selectively inhibit the cleavage of APP. Such an inhibitor would represent a new class of AD therapeutics: APP-selective BACE1 inhibitors (ASBIs). As we previously described, the hypothesized mechanism for ASBI selectivity is binding to APP and inhibition of APP binding to the BACE1 active site, but not that of other substrates, resulting in potent inhibition of APP cleavage by BACE1 compared to other substrates [26].

Development of an ASBI may not only provide a potential new treatment for AD and the condition that often precedes it, Mild Cognitive Impairment (MCI) due to AD, but may do so with reduced off-target effects as compared to BACE1 inhibitors that have been clinically tested and are not selective for APP.

The first step in our approach to discover ASBIs was to screen for BACE1 inhibitors by using a maltose binding protein (MBP)-conjugated-APPC125 substrate-based assay. In an initial screening of a clinical compound library, we identified flavonoids that acted as ASBIs in cell models and one (galangin) that showed increased brain levels when delivered to mice as a pro-drug, as reported in Descamps et al. [26].

We describe herein that, as a result of our continued screening efforts, we identified the hit phenytoin as a weak inhibitor of BACE1 cleavage of MBP-APPC125 and, through medicinal chemistry optimization efforts, discovered an APP-selective fluoro aminohydantoin (FAH) BACE1 inhibitor series. The lead candidate in the series is the APP selective small molecule FAH65 and its active enantiomer FAH65(-), which displays potent inhibition of BACE1 *in vitro* and *in vivo*, and elicits improvement in memory in an AD mouse model after oral treatment.

## Materials and methods

### P5-P5′ assay

For the P5-P5′ BACE1 inhibition assay, 4 ​μL of assay buffer was added to each well, followed by 2 ​μL of BACE1 diluted in assay buffer to 7.5 ​ng/μL. Then, 2 ​μL of inhibitor, at concentrations along an 11 ​pt. two-fold dilution series starting at 10 ​μM, or 0.5 ​μM, were added to appropriate wells and incubated for 30 ​min at room temperature. For all dilutions of inhibitor, drug stock solutions of 10 ​mM in DMSO were diluted to 50 ​μM in water; subsequent dilutions were in 0.5 ​% DMSO. Afterwards, 2 ​μL of fluorogenic P5-P5′ BACE1 substrate, diluted to 50 ​μM in assay buffer, were added to each well and the signal generated was read every 30 ​min at 25 ​°C for 2 ​h.

### SEAP-NRG assay

A cDNA construct encoding a human placental secreted alkaline phosphatase (SEAP)-NRG1 (pAPtag5-NRG1-β1) fusion protein was transfected in human embryonic kidney (HEK293) cells in a 6-well format with or without full-length wild-type BACE1 using Lipofectamine 2000 (Invitrogen) as described previously [27]. After transfection, the medium was replaced with DMEM containing the test compound at 10, 5, 1, 0.1, and 0.01 ​μM as well as 10 ​% heat-inactivated fetal bovine serum (FBS), then incubated for 24 ​h. SEAP activity was measured in the conditioned medium. For alkaline phosphatase activity measurements, 200 ​μL of reaction solution (0.1 ​M glycine, pH 10.4, 1 ​mM MgCl_2_, 1 ​mM ZnCl_2_ containing 1 ​mg/mL 4-nitrophenyl phosphate disodium salt hexahydrate, Sigma) were added to 20 ​μL of the conditioned medium. The absorbance was read at 405 ​nm.

### PSGL1 assay

A cDNA construct encoding a human placental secreted alkaline phosphatase (SEAP)-PSGL1 (pAPtag5-PSGL1-β1) fusion protein was transfected in human embryonic kidney (HEK293) cells in a 6-well format with or without full-length wild-type BACE1 using Lipofectamine 2000 (Invitrogen). After 6–8 ​h transfection, the medium was replaced with DMEM containing the test compound at 10, 5, 1, 0.1 and 0.01 ​μM and 10 ​% heat-inactivated fetal bovine serum (FBS), and incubated for 24 ​h. SEAP activity was measured in the conditioned medium. For alkaline phosphatase activity measurements, 200 ​μL of reaction solution (0.1 ​M glycine, pH 10.4, 1 ​mM MgCl_2_, 1 ​mM ZnCl_2_ containing 1 ​mg/mL 4-nitrophenyl phosphate disodium salt hexahydrate, Sigma) were added to 20 ​μL of the conditioned medium. The absorbance was read at 405 ​nm at 60 ​min.

### Cathepsin D assay

The effects of candidate ASBIs on cathepsin D (CatD) activity was assessed using the R&D Systems CatD activity assay kit (Catalog #: 1014-AS and ES001) according to the manufacturer's instructions. For the assay, 4 ​μL of assay buffer was added to each well, followed by 2 ​μL of CatD diluted in assay buffer to 5 ​ng/uL. Then, 2 ​μL of inhibitor, at concentrations along an 11 ​pt. two-fold dilution series starting at 50 ​μM, were added to appropriated wells and incubated for 30 ​min at room temperature. For all dilutions of inhibitor, drug stock solutions of 10 ​mM in DMSO were diluted to 50 ​μM in water; subsequent dilutions were in 2.5 ​% DMSO. Afterwards, 2 ​μL of fluorogenic CatD substrate, diluted to 50 ​μM in assay buffer, were added to each well, after which the signal generated was read every 30 ​min at 25 ​°C for 1hr.

### Plasmids

The pAPtag5-NRG1-β1 construct was kindly provided by Dr. Carl Blobel [28]. The BACE1 construct was a gift from Dr. Michael Willem and Dr. Christian Haass [27].

### Medicinal chemistry and testing of analogs

Our exploratory medicinal chemistry efforts focused on the phenytoin hit to see if we could generate analogs with enhanced potency. The medicinal chemistry efforts (planned to be published elsewhere) involved conversion of the hydantoin scaffold to an aminohydantoin that led to our potent ASBI FAH65. Analogs were assessed in the MBP-APPC125, p5-p5′, and substrate selectivity assays as described.

### Synthesis

The methods for FAH analog synthesis are described in the Supplementary Materials.

### CHO-7W and -7W^Swe^ cell culture

The Chinese hamster ovary (CHO) cell line overexpressing human AβPP (7W) stably transfected with human APP or overexpressing APP with the Swedish (Swe) K595 ​N/M596L mutation were incubated with FAH compounds diluted to 1 ​μM overnight. Culture Media was collected and frozen. Media was assayed using an AlphaLISA for Aβ1-42 (Perkin Elmer catalog # AL276C), sAPPα (R&D Systems catalog # AF1168 conjugated with Perkin Elmer acceptor beads catalog # 6772001 ​+ ​2B3 antibody from IBL catalog # 11088 biotinylated), and sAPPβ (Perkin Elmer AL276-acceptor ​+ ​IBL catalog # 18957 biotinylated). For the assay, 2 ​μL of diluted or undiluted sample was added to a 384 well plate, followed by 2 ​μL of the respective antibody mixture. This was incubated for 1 ​h. Afterwards, 2 ​μL of donor beads was added to each well, followed by a 30-min incubation in the dark, after which the signal was read.

### Pharmacokinetics (PK)/Pharmacodynamics (PD) in mice and rats

Wildtype or non-transgenic (NTg) C57Bl6 mice or Sprague-Dawley rats were dosed by oral (gavage) or subcutaneous (SQ) injection at 10 or 30 ​mg/kg, and animals were euthanized 1, 2, 6, and 8 ​h after administration by ketamine/xylazine over-anesthesia, in rats, CSF was also collected, followed by transcardial collection of blood for isolation of plasma and saline perfusion. Brain tissue was collected post-mortem for assessment of compound levels and biomarkers.

Analyses of compound levels in PK studies were performed in the UCLA Pasarow Mass Spectrometry Lab (PMSL; Julian Whitelegge, Ph.D., Director). Tissues were homogenized in a bead beater using 5 ​vol of ice-cold 80 ​% acetonitrile (1/5; mg of brain/μL of 80 ​% ACN). Plasma analytes were extracted using 4 ​vol of ice-cold acetonitrile (1/4; μL of plasma/μL of 100 ​% ACN). Solutions were clarified by centrifugation (16,000×*g*, 5 ​min) and the supernatants were transferred to new tubes and lyophilized. Samples were reconstituted in 100 ​μL of 50/50/0.1 (Water/Acetonitrile/Formic Acid) prior to analysis via liquid chromatography-tandem mass spectrometry (LC-MS/MS).

An internal standard (IS) was added to every sample to account for compound loss during sample processing. Standards were made in drug naïve plasma and tissue lysates with increasing amounts of analyte (S1, S2: 0 pmol/S3, S4: 1 pmol/S5, S6: 10 pmol/S7, S8: 100 ​pmol, S9, S10: 1000 ​pmol). The standard curve was made by plotting the known amount of analyte per standard vs. the ratio of measured chromatographic peak areas corresponding to the analyte over that of the IS (analyte/IS). The trendline equation was then used to calculate the absolute concentrations of each compound in plasma and tissue.

The targeted LC-MS/MS assay was developed using the multiple reaction monitoring (MRM) acquisition method on a 6460 triple quadrupole mass spectrometer (Agilent Technologies) coupled to a 1290 Infinity HPLC system (Agilent Technologies) with a Phenomenex analytical column (Kinetex 1.7 ​μm C18 100 ​Å 100 ​× ​2.1 ​mm). The HPLC method utilized a mixture of solvent A (99.9/0.1 Water/Formic Acid) and solvent B (99.9/0.1 Acetonitrile/Formic Acid) and a gradient was use for the elution of the compounds (min/%B: 0/1, 3/1, 19/99, 20/1, 30/1). Two fragment ions were monitored at specific LC retention times to ensure specificity and accurate quantification in the complex biological samples. The normalized chromatographic peak areas were determined by taking the ratio of measured chromatographic peak areas corresponding to the compound over that of the internal standard (Analyte/IS).

The T_max_, C_max_, brain-to-plasma ratios and brain levels were then calculated using PK Solutions software (SummitPK).

### PK/PD study of FAH65E(-) in ApoE4-TR:5xFAD mice

A PK/PD study of FAH65E(-) hydrochloride was performed using ApoE4-TR:5xFAD mice that express APOE4 under the control of the endogenous mouse APOE promoter crossed to 5xFAD mice (Tg6799) which co-express five FAD mutations (APP K670 ​N/M671L ​+ ​I716V ​+ ​V717I and PS1 M146L ​+ ​L286V) under the control of the neuron-specific mouse Thy-1 promoter [29,30]. The resulting mice, on a 97 ​% C57Bl/6J and 3 ​% SJL background, are homozygous for APOE4 and hemizygous for the 5XFAD transgenes. Both male and female mice 6–8 months of age were used. Mice received two doses (a.m. and p.m.) of 30 ​mg/kg FAH65E(-) by oral gavage on day 1; on day 2, after a single dose (a.m.) mice were euthanized at 1, 2, 4 and 6 ​h, n ​= ​3 per time point. FAH65 and sAPPβ levels in brain were determined in brain tissue by LC-MS (see Supplementary Methods) and ELISA , respectively.

### Efficacy testing in B254 mice

B254 mice are an AD model comprising expression of human APP with Swedish and Indiana mutations along with a D664A substitution and endogenous murine tau, as described in Galvan et al. [31]. In our *in vivo* studies, we detected impairment in learning and memory in B254 D664A mice at 5–6 months of age (end of chronic treatment) in the novel location and novel object spatial memory testing paradigms. We note that previous studies have reported conflicting findings for such impairments when using in the Morris Water Maze. In the earliest reports [31,35], while B254 mice showed similar Aβ production at 3–4 months and earlier plaque deposition (starting at ​∼ ​6 months) as J20 (PDAPP) mice (plaque deposition after 9 months), which were considered a control with an unaltered D664 sequence and functional caspase cleavage, behavioral abnormalities in MWM testing were not observed at 12 months. Further study suggested an akinetic phenotype that could be overcome by swim training may have been the cause of these different findings [36]. In a later study performed by a different group [37], it was reported that at both 2–3 or 5–7 months of age B254 and J20 mice had similar abnormalities relative to non-transgenic mice in spatial and nonspatial learning and memory. Our findings were in alignment with the latter studies.

Male and female 4-5 month-old B254 mice in the subchronic and chronic treatment were administered FAH65 via pipette feeding by the method of Atcha et al. [32] at a dose of 30 ​mg/kg BID (60 ​mg/kg/day). Mice were weighed before the first dose for the calculation of volume to be administered. The formulation used comprised FAH65 hydrochloride as a 40 ​mg/mL solution in water plus 30 ​mg/mL in strawberry syrup.

In the pilot subchronic study, mice were treated for 10 days, and in the follow-up study, for 26 days. One week before the end of the study, mice in the pilot study underwent Novel Object Recognition (NOR) memory testing, and mice in the follow-up chronic study underwent both NOR and Novel Location Recognition (NLR) testing as described elsewhere [33,34]. Briefly, mice are introduced to two identical, parallel and evenly objects in an opaque 30 ​× ​40 ​cm arena (box without a lid) with navigation marks on the wall, for 10 ​min. For Novel Location Recognition (NLR), after a 2.5-h interval, the mouse is returned to the arena wherein one identical object has been moved diagonally. The time spent and number of discreet interactions with the objects in the original location and in the new location over 10 ​min is recorded. The mouse is then removed from the arena, and after an additional 2.5-h interval, for the Novel Object Recognition (NOR) phase, is returned to the arena wherein the objects are in the original position, but one is a new, different object. The time spent and number of discreet interactions with the objects in the original location and in the new location over 10 ​min is recorded. The novelty preference was calculated by dividing total interactions (or time) by interactions (or time) with the object in the novel location or the novel object.

All *in vivo* animal experiments using mice or rats described herein were carried out in strict accordance with good animal practice according to the U.S. National Institutes of Health (NIH) recommendations. All procedures for animal use were approved by the Animal Research Committee (ARC) at the University of California at Los Angeles (UCLA) and under an approved ARC protocol and comply with the ARRIVE guidelines.

### Biomarker analyses

For *in vivo* studies, Individual tissues were weighed and sonicated on ice with AlphaLISA Lysis Buffer complemented with HALT (Fisher catalog # 78446) to give a 10 ​% W/V sonicate. Total protein concentration was assessed by BCA assay (Fisher catalog # 23227) following manufacturer recommendations.

### sAPPβ

For the ELISA assay to assess sAPPβ levels in brain an IBL ELISA (kit #27733) specific for sAPPβ with the Swedish (K595 ​N/M596L) mutation [12] that is present in the APP transgene of the mouse model used following the kit instructions. Briefly, 499 ​μL of EIA buffer was added to individual tubes for each sample, 1 ​μL of sample added, vortexed and centrifuged. To each assay plate well, 100 ​μL of sample was added, and the covered plate incubated overnight at 4 ​°C. The plate(s) was washed 4 times with 350 ​μL/well wash buffer, then 100 ​μL/well of labeled antibody added followed by incubation for 30 ​min at 4 ​°C. After five washes, 100 ​μL chromogen solution was added to each well and incubated for 30 ​min at RT in the dark. Stop solution (100 ​μL/well) was then added and the plate read at 450 ​nm.

### βCTF

The BACE1 cleavage product βCTF was detected by AlphaLISA (Perkin Elmer) Amyloid Beta kit (Cat # AL275C) modified by replacing the anti-Aβ acceptor beads (Cat # AL275AC) specific for the C-terminus of Aβ40 with anti-Aβ acceptor beads from the AL202 kit (Cat #AL202AC) having the 4G8 antibody. For the assay, 2 ​μL of diluted sample was added to a 384 well plate, followed by 2 ​μL of the antibody mixture. This was incubated for 1hr. Afterwards, 2 ​μL of donor beads was added to each well, followed by a 30-min incubation in the dark, after which the signal was read.

### sAPPα

sAPPα was assessed by AlphaLISA comprising antibody AF1168 (R&D Systems catalog # AF1168) conjugated with Perkin Elmer acceptor beads (catalog # 6772001) and 2B3 antibody from IBL catalog # 11088 biotinylated and streptavidin donor beads. For the assay, 2 ​μL of diluted or undiluted sample was added to a 384 well plate, followed by 2 ​μL of the respective antibody mixture. This was incubated for 1 ​h. Afterwards, 2 ​μL of donor beads was added to each well, followed by a 30-min incubation in the dark, after which the signal was read.

### Aβ1-42

For assessment of Aβ 1-42 in brain, tissues were weighed and sonicated in freshly prepared 5 ​M Guanidine (Gdn)-HCl in 50 ​mM Tris–NCl pH 8.0 at 20 ​% weight/volume. Samples were rotated for 2–3 ​h at room temperature after sonication and typically frozen stored before use. An Invitrogen ELISA kit for 1-42 was used according to the manufacturer's instructions.

### Total tau and p-tau

Phospho-tau and total tau were assessed by Perkin-Elmer AlphaLISA kits AL271 (phosphorylation at Ser202 and Thr205) and AL3136 (total tau). The assays were run as described above for the sAPPα AlphaLISA.

### In supplementary methods

The BACE2, MBP-APPC125, ADME-T (kinetic solubility, plasma stability, liver microsome stability, parallel artificial membrane permeability assay/PAMPA, plasma protein/human serum albumin, HSA, binding, and brain tissue binding), FAH binding to APP and hERG assays are described in Supplementary Methods. Also in Supplementary Methods are descriptions of the testing of FAH65E(-) in HEK-APP-GFP cells, Meso Scale Discovery (MSD) assessment of inflammatory biomarkers GFAP and IP-10, as well as Aβ in mouse brain, *in vivo* assessment of FAH65 versus verubecestat (Veru) in rats, Super Fluid Critical (SFC) separation of FAH65 enantiomers and assessment of their optical rotation, and syntheses/analyses of FAH analogs.

## Results

### Screening and analog generation by medicinal chemistry identifies lead FAH65

We identified a known anti-epilepsy hydantoin drug, phenytoin, as a screening hit in the MBP-APPC125 BACE1 cleavage screening assay [26]. Initial exploratory medicinal chemistry on the phenyl rings (A and B-rings) of the hydantoin hit revealed no significant improvement in BACE1 inhibition. Further modification of the hydantoin scaffold to an aminohydantoin led to a micromolar BACE1 inhibitor fluoroaminohydantoin, FAH1, with A-ring fluorine substitutions that inhibited Aβ42 in cells and was brain permeable. Further modification of the B-ring led to a more potent low micromolar inhibitor, FAH3, that also inhibited Aβ in cell models and had brain permeability, but also showed selectivity for APP cleavage by BACE1 versus other substrates such as NRG1. The BACE1 IC50s in the cell-free P5-P5′ activity assay for FAH1 and FAH3 were ∼10 ​μM and ∼1 ​μM, respectively (See Supplementary Fig. S1A). In Chinese Hamster Ovary cells expressing wildtype human APP (CHO-7W), Aβ42 was decreased by 5 ​μM FAH1 and by 1 ​μM FAH3.

**Fig. 1:**
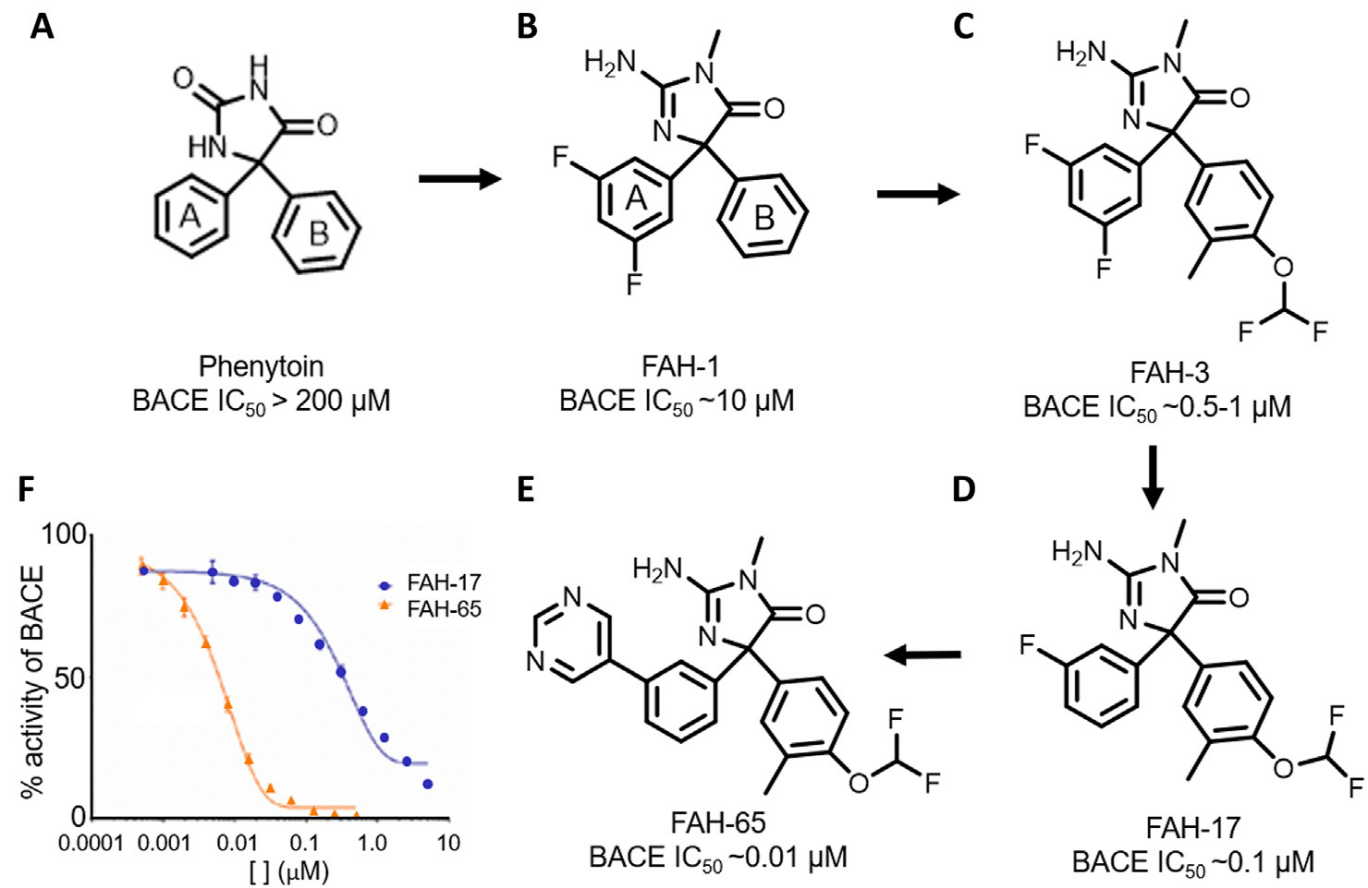
*Analog structures and inhibition of BACE*. The structures of (A) phenytoin, (B) FAH1, (C) FAH3, (D) FAH17 and (E) FAH65 are shown, as well as the (F) dose-response curve for FAH17 and FAH65 in the P5-P5′ BACE inhibition assay.

Interestingly, removal of a fluorine from the A-ring led to a more potent analog, FAH17 (IC50 ∼ 0.3 ​μM) and further replacement of the fluorine with a pyrimidine ring gave lead compound FAH65 (IC50 ∼ 0.01 ​μM–0.03 ​μM) in the P5-P5′ assay (Fig. 1, Supplementary Fig. S1A). Details of the structure-activity strategy and analog synthesis effort will be described in a future manuscript.

### FAH65 selectively targets BACE1 and inhibition of APP cleavage

**Fig. 2:**
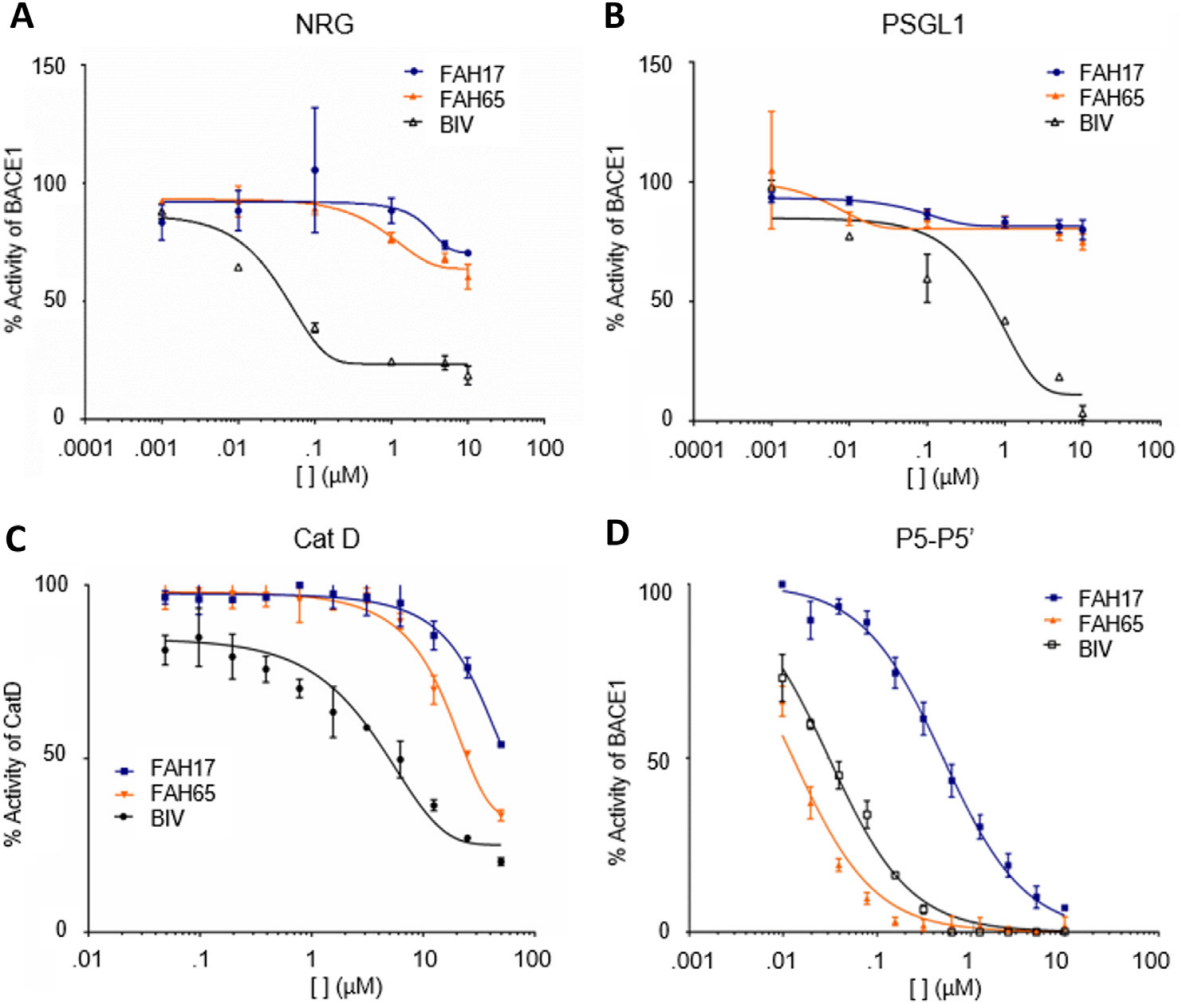
*FAH65 selectivity*. Dose-response curves for FAH17 and FAH65 as compared to BACE1 inhibitor BACEIV are shown for the substrates (A) NRG1 and (B) PSGL1 in cell assays. Dose-response curves for inhibition of (C) cathepsin D (Cat D) and (D) BACE in the P5-P5′ assay are shown.

Ongoing selectivity monitoring using a SEAP-neuregulin 1 (NRG1) assay demonstrated the analogs were selective and did not inhibit BACE1 cleavage of NRG1. FAH3 elicited 50 ​% inhibition of BACE1 cleavage of APP, but no SEAP-NRG cleavage, at ∼1 ​μM. Similarly, neither FAH17 nor FAH65 show potency in cleavage of either NRG1 or PSGL1 (both EC_50_s ​> ​10 ​μM) vs. BACE1-APP (IC_50_s of 0.3 ​μM and 0.01 ​μM, respectively) in cell-free assays as compared to the inhibitor BACEIV that has little selectivity for APP (BACE1-APP IC_50_ ∼0.04 ​μM) vs BACE1-NRG1 or PSGL1 (both EC_50_ ∼0.05 ​μM) (Fig. 2A and B, respectively). In comparison, clinically-studied BACE1 inhibitors Veru (MK-8931) and lanabecestat (Lana; AZD3293) show no selectivity for BACE1-APP (IC_50_s of 0.002 and 0.004 ​μM, respectively) vs. BACE1-NRG1 (EC_50_ of <0.01 ​μM for both); with substrate selectivity: NRG1/APP IC_50_ of >200 for FAH65 and <2.5 for both Veru and Lana (Supplementary Table S1).

FAH17 and FAH65 were also found to be selective for BACE1 as the target enzyme. Neither analog significantly inhibited cathepsin D (Cat D) activity (Fig. 2C), and lead FAH65 did not inhibit BACE2 activity compared to BACE-IV (Supplementary Fig. S2); whereas both inhibit P5-P5′ cleavage by BACE 1 (Fig. 2D). The FAH65 IC_50_ for Cat D, BACE2, and BACE1 are ∼47 ​μM, >1 ​μM and ∼0.01 ​μM, respectively.

### FAH65 inhibits sAPPβ and Aβ1-42 production in APP-expressing cells *in vitro*

**Fig. 3:**
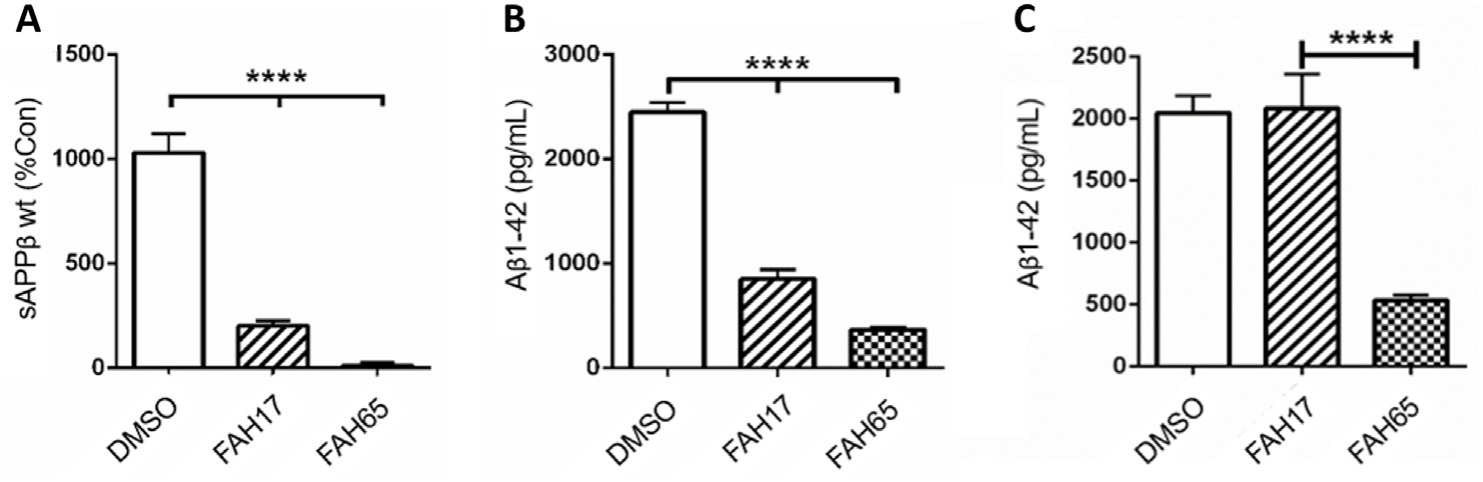
*sAPPβ and Aβ1-42 production in CHO-7W and CHO-Swe cells*. Levels of sAPPβ (A) and (B) Aβ1-42 in CHO-7W wildtype APP, and (C) Aβ1-42 in APP Swe cell lysates after 24-h treatment at 1 ​μM are shown. Data graphed as the mean and SEM. Statistical analysis performed using one-way ANOVA and Dunnett's post-hoc analysis.

Both FAH17 and FAH65 display the ability to decrease production of BACE1 cleavage product soluble APPβ (sAPPβ) and amyloid-β 1-42 (Aβ1-42) in Chinese hamster ovary cells expressing wildtype human APP (CHO-7W) (Fig. 3A and B, respectively), with an EC50 for sAPPβ reduction by FAH65 of >90 ​%. Only FAH65 significantly decreases Aβ1-42 in CHO cells that express APP with the Swedish (Swe) K595 ​N/M596L mutation (Fig. 3C). Cells expressing APP with the Swedish mutation were tested in anticipation of *in vivo* studies in AD model mice that express human APP-Swe.

### Pilot APP binding experiments of the FAH analogs by surface plasmon resonance (SPR)

The interaction of FAH3, 17 and 65 were evaluated using the SPR protocol described previously in Descamps et al., [26]. The FAHs showed varying interaction with APP, when compared to the BACEIV inhibitor (Supplementary Table S2). The binding to APP may contribute to the selectivity seen with the FAHs for BACE1 cleavage of APP substrate relative to NRG1 and PSGL1 substrate.

### FAH65 displays favorable physiochemical properties and brain permeability in mice

FAH65 was determined to have kinetic solubility at 88 ​μM in water, plasma stability at t_1/2_ > 180 ​min and, in the Parallel Artificial Membrane Permeability (PAMPA) assay, a Pm of 1.09, predicting it would be brain-permeable. Its half-life in liver microsomes is ∼38 ​min and it binds human serum albumin (HSA) with a free unbound (F_u_) of 0.44 ​%. In brain tissue, the F_unbound_ was 3.1 ​% for (see Supplementary Methods and Supplementary Table S3). In the human ether-a-go-go-related gene (hERG) testing, FAH65 showed potassium channel binding below 50 ​%, ranging from 38.7 to 41 ​% at 10 ​uM (IC_50_ ​> ​10 ​μM).

**Fig. 4:**
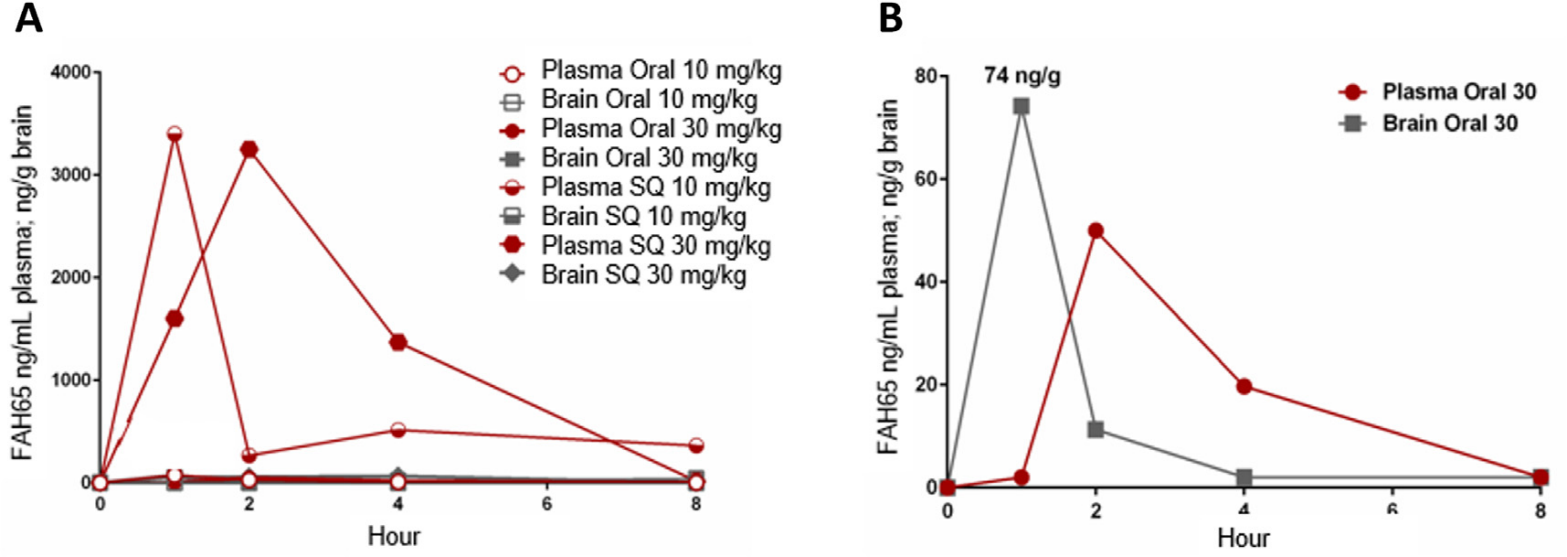
*Pharmacokinetics of FAH65 in mice*. (A) Plasma and brain levels of FAH65 at 10 and 30 ​mg/kg 1, 2, 4 and 8 ​h after delivery by either the oral or subcutaneous (SQ) routes are shown. (B) Plasma and brain levels of FAH65 shown with an expanded y-axis scale are above IC_50_ after oral delivery at 30 ​mg/kg.

After subcutaneous (SQ) delivery of FAH65 to mice at 10 or 30 ​mg/kg, compound levels over 3000 ​ng/mL were observed in plasma (Fig. 4A). After oral delivery of FAH65 at 30 ​mg/kg, the plasma C_max_ of ∼50 ​ng/mL was seen at 2 ​h and the brain C_max_ of 74 ​ng/g at 1 ​h (Fig. 4B).

### Pilot PK/PD comparison of FAH65 and Veru in rats

A pilot PK/pharmacodynamic (PD) study was performed in rats to compare FAH65 with known BACE inhibitor Veru [23]. After oral gavage delivery at 30 ​mg/kg, the C_max_ for brain of 182 ​ng/g for FAH65 (Supplementary Fig. S3A) and 157 ​ng/ng for Veru (Supplementary Fig. S3B) were observed at 2 ​h post-delivery, showing similar brain permeability. In cerebral spinal fluid (CSF), rat APP BACE1 cleavage product sAPPβ was the lowest at 2 ​h post-delivery of Veru and 3 ​h post-delivery for FAH65 (1- and 2-h samples were not available for assay) and were lower with FAH65 (Supplementary Fig. S3C). In brain (medial cortex), sAPPβ was lowest 6 ​h post-delivery of FAH65 (Supplementary Fig. S3D). In addition to sAPPβ, BACE1 cleavage of APP produces the β C-terminal fragment (βCTF), which can then be cleaved by γ-secretase to produce Aβ1-40 and/or Aβ1-42. Aβ1-40 was also lower in CSF from FAH65-treated as compared with Veru-treated rats (Supplementary Fig. S3E). Aβ1-42 in brain varied with FAH65 and Veru treatment (Supplementary Fig. S3F). Inhibition of APP cleavage by BACE1 likely provides more available substrate for α-secretase cleavage and sAPPα generation; sAPPα levels in brain medial cortex were higher with FAH65 (Supplementary Fig. S3G).

### FAH65 improved working memory *in vivo* in a murine model of AD

**Fig. 5:**
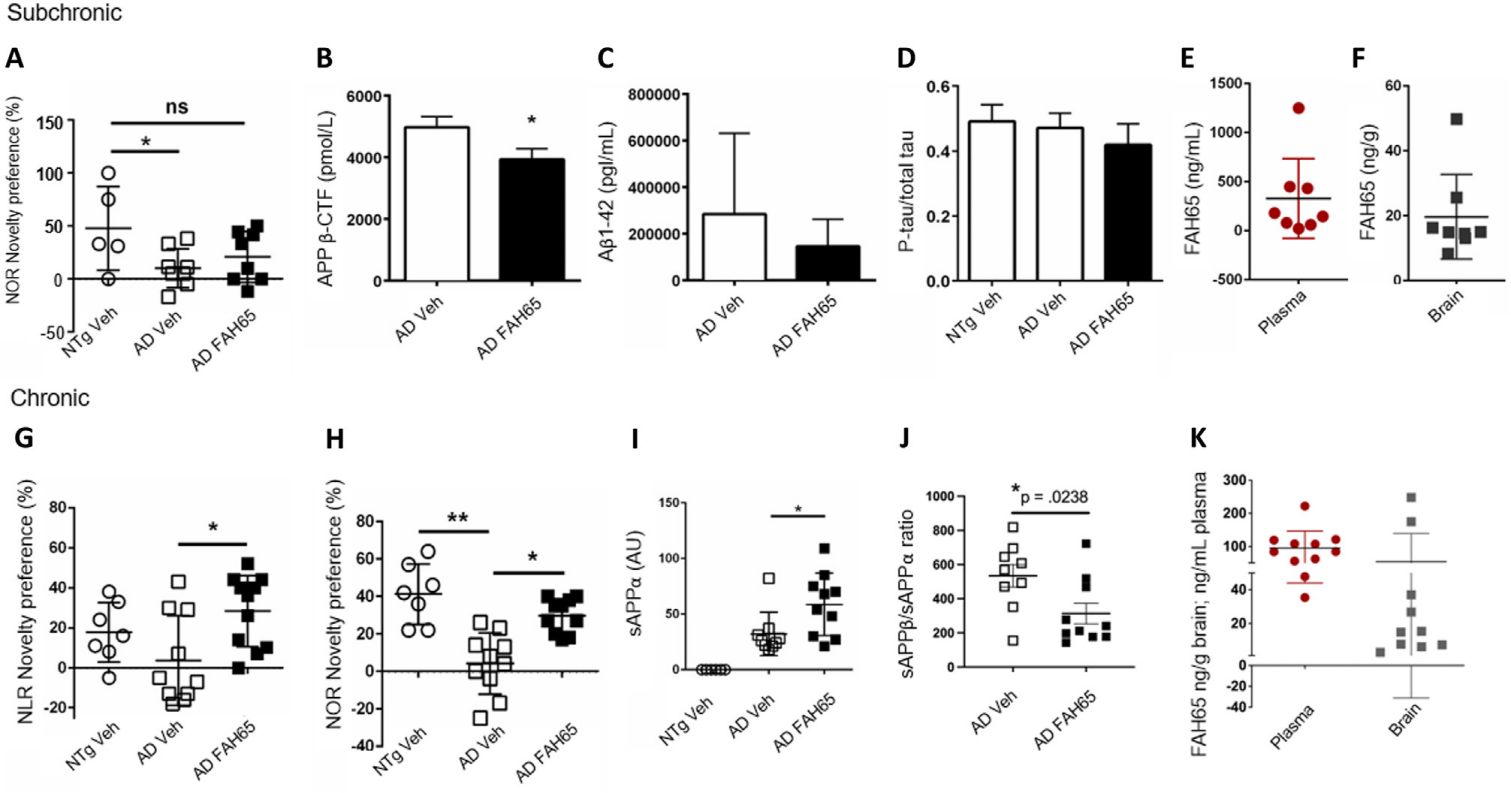
*FAH65 efficacy in AD model mice*. Pilot subchronic study (A) novelty preference in the Novel Object Recognition (NOR) testing paradigm and brain levels of (B) APP β-CTF, (C) Aβ 1-42, and (D) the p-tau/total tau ratio, (E) plasma and (F) brain levels of FAH65 in non-transgenic (NTg; n ​= ​5), AD model vehicle-treated (AD Veh; n ​= ​8), and FAH65-treated (AD FAH65; n ​= ​8) AD model mice are shown. Follow-up chronic study (G) novelty preference in Novel Location Recognition (NLR), and (H) NOR are shown, as well as brain levels of (I) sAPPα, and (J) the sAPPβ/sAPPα ratio. (K) Plasma and brain levels of FAH65 are shown. NTg Veh (n ​= ​7), AD Veh (n ​= ​9), and AD FAH65 (n ​= ​11) mice; both studies used 4-5 month-old female and male mice. Data graphed as the mean and SEM. Statistical analysis performed using one-way ANOVA with Dunnett's group comparisons; ∗p ​< ​0.05 and ∗∗p ​≤ ​0.01.

In the pilot subchronic study (n ​= ​8) of oral 30 mkd FAH65 for 10 days in B254 mice, the performance of FAH65-treated mice in the Novel Object Recognition (NOR) testing paradigm [34] was not significantly increased as compared to vehicle-treated mice (Fig. 5A). Target engagement in the brain was evidenced by the significant decrease in APP β-CTF in brain tissue of FAH65-treated as compared to vehicle-treated mice (Fig. 5B). While the means for Aβ 1-42 and the p-tau/t-tau ratio were lower in FAH65 vs. vehicle-treated mice, the differences were not significant (Fig. 5C and D). Plasma and brain levels of FAH65 2 ​h after the last dose of FAH65 on the last day of treatment (time of euthanasia) are shown in Fig. 5E and F, respectively; FAH65 showed modest brain levels, with a mean of ∼ 20 ​ng/g in brain.

Levels of inflammatory biomarkers for astrocytic gliosis (GFAP) and microglial activation (IP-10), as well as Aβ1-38, Aβ1-40, Aβ1-42, and the Aβ1-42/1-40 ratio (Supplementary Fig. S4A–F, respectively), were assessed by MSD in brain tissue from mice in the subchronic study, this was a limited set of neuroinflammation markers but no significant differences between vehicle- and FAH65-treated B254 mice were observed.

In the follow-up chronic study (n ​= ​11) of oral 30 mkd FAH65 for 26 days, a significant increase in novelty preference was observed in both Novel Location Recognition (NLR) and NOR for FAH65- vs. vehicle-treated mice (Fig. 5G and H). sAPPα, product of α-secretase cleavage of APP that may be expected to increase with greater substrate availability due to BACE1 inhibition, was significantly higher (Fig. 5I) and the sAPPβ/sAPPα ratio representing the balance of cleavage by these competing pathways, was significantly lower (Fig. 5J) in brain tissue of FAH65-treated mice as compared to vehicle-treated mice, supporting target engagement. Brain levels of sAPPβ and Aβ1-42 were also assessed in the study and were found to be highly variable amongst mice and not significantly different for Veh vs. FAH65-treated mice. The mean brain level of FAH65 was ∼50 ​ng/g 2 ​h after last day dosing (Fig. 5K).

### Separation, configuration and testing of the active enantiomer of FAH65

**Fig. 6:**
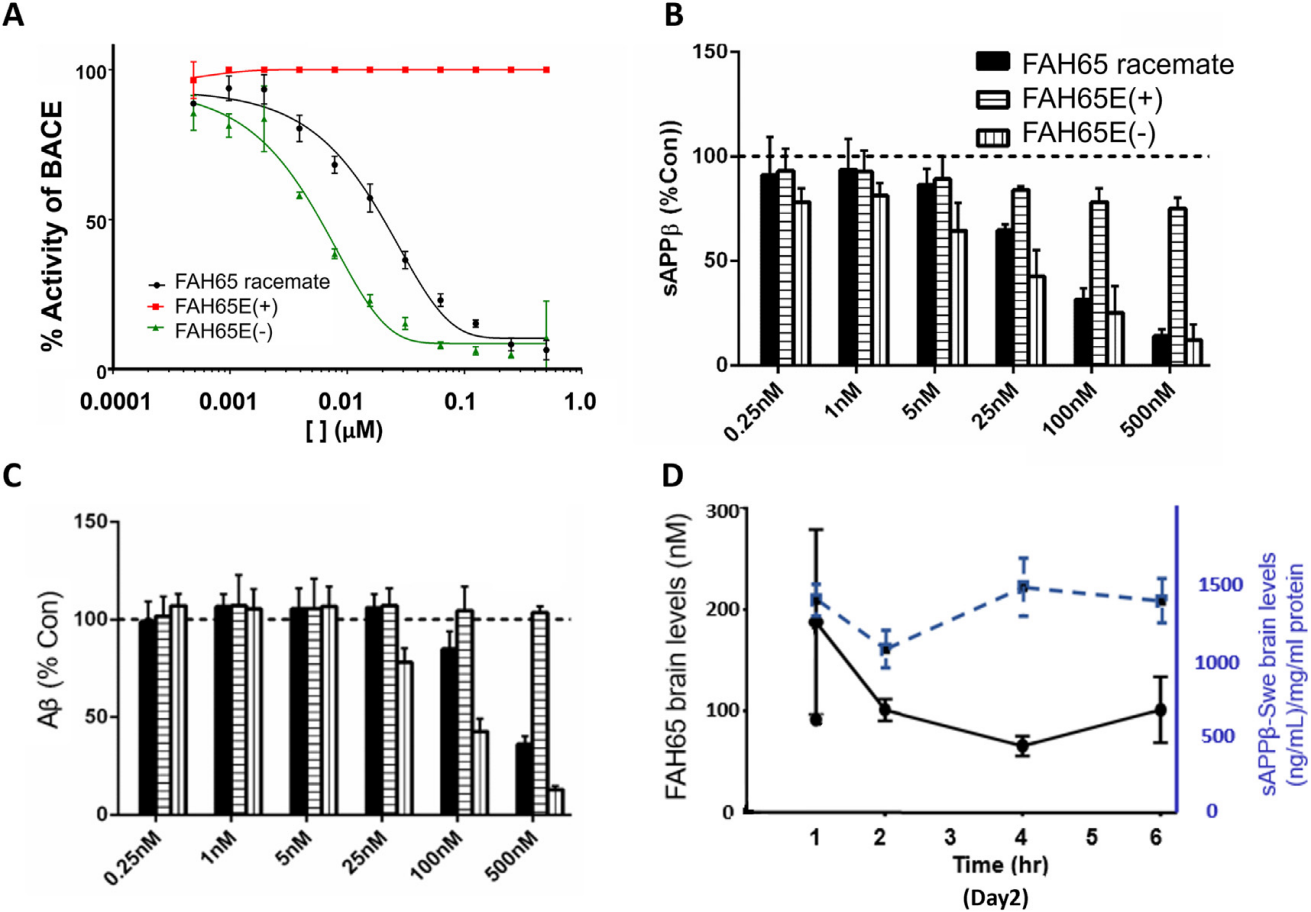
*Inhibitory activity and brain permeability of FAH65E(-)*. Shown are (A) dose-response curves for FAH65 racemate and the FAH65E(+) and (-) enantiomers in the P5-P5′ assay and (B) sAPPβ and (C) Aβ1-42 in CHO-7W cells after treatment with increasing concentrations of FAH65 racemate and enantiomers. Legend in B also applies to C. Data graphed as the mean and SEM. (D) FAH65E(-) (black line) and sAPP*β* (blue dashed line) levels in brain from ApoE4TR-5XFAD mice after oral delivery of 30 ​mg/kg FAH65E(-) doses are shown. PK-PD study design did not include 0 h timepoint untreated mice but included time points 1, 2, 4 and 6 ​h after last dose on Day 2. N ​= ​3 mice per time point.

The (+) and (-) enantiomers of FAH65 were separated using supercritical fluid chromatography as described in Supplementary Methods and shown in Supplementary Fig. S5. The absolute configuration of the active enantiomer was determined to be the *S*-configuration based on the X-ray crystallography of the inactive (+) enantiomer using procedure described in Supplementary Methods. The enantiomers underwent assessment for relative BACE1 inhibition. In the P5-P5′ assay, the (-) enantiomer of FAH65, FAH65E(-), shows high activity (IC_50_ ∼0.005 ​μM), whereas the FAH65(+) enantiomer is inactive (Fig. 6A). FAH65 E(-) is also active in the MBP-APPC125 assay (Supplementary Fig. S6). FAH65E(-) induced a dose-response reduction in sAPPβ in CHO-7W cells at concentrations of 5 ​nM and higher with an EC_50_ ∼25 ​nM (Fig. 6B), and in Aβ1-42 at concentrations of 25 ​nM and higher (Fig. 6C).

To assess FAH65E(-) effects in a human cell line, HEK293T cells stably expressing full-lenght (FL) APP with an N-terminal GFP label were treated with FAH65E(-), BACEIV and Verubecestat (Supplementary Fig. S7). Decreased BACE1 cleavage of FL APP was anticipated to result in a greater fluorescent signal intensity, because more FL APP should remain associated with the cells. As shown in Supplementary Fig. S7B, the signal intensity was significantly greater with FAH65E(-) at 1 μM as compared to DMSO (Supplementary Fig. S7C). Representative GFP signal in microscopic images of HEK-APP cells is shown in Supplementary Fig. S8.

The crystal structure for the inactive FAH-65(+) is shown in Supplementary Fig. S9.

In ADME-T testing (see Supplementary Methods and Supplementary Table S3), FAH65E(-) displayed favorable physiochemical properties to be an orally bioavailable therapeutic *in vivo*, including kinetic solubility at 79 ​μM, potential for blood-brain barrier (BBB) permeability at 1.01 Pm in PAMPA, and plasma stability at t_1/2_ ​> ​180 ​min. It has a moderately short half-life in liver microsomes (49 ​min) and bound to human serum albumin (HAS) at relatively high levels (*F*_*u*_ 0.49 ​%). The *F*_*u*_ was 5.4 ​% for brain tissue.

*In vivo* in a pharmacokinetic/pharmacodynamic (PK-PD) study was done in ApoE4TR:5xFAD mice. FAH65E(-) was observed to be orally brain bioavailable, reaching a maximum brain concentration (C_max_) of 188 ​nM at 1 ​h after oral administration of the last of three doses (two on day 1, one on day 2) at 30 ​mg/kg to ApoE4-5XFAD mice (Fig. 6D, black line). On day 2 the brains were collected at 1, 2, 4 and 6 ​h after the last dose. In these mice, sAPPβ levels decreased at 2 ​h after oral administration of the last dose (Fig. 6D, blue dashed line), suggesting a pharmacodynamic correlation between brain C_max_ levels and sAPPβ, further supporting target engagement after oral administration of FAH65E(-).

## Discussion

We describe herein our discovery of ASBI lead candidate FAH65 and its active enantiomer FAH65E(-) through screening, optimization by exploratory medicinal chemistry, analog synthesis and testing. The lead candidate FAH65 is highly selective for BACE1 as the target enzyme and APP as the substrate. *In vitro* studies reveal the selectivity and potency of FAH65, and *in vivo* studies in AD model mice provide evidence that FAH65 decreases APP cleavage product sAPPβ in brain tissue and increases trophic, neurite-supporting cleavage product sAPPα, along with significant improvements in memory in the NOR/NLR testing paradigms after oral treatment at 30 mkd for 26 days.

In the pilot study in rats, FAH65 displayed good brain penetrance and target engagement, lowering sAPPβ and Aβ1-40/42, with a concomitant increase in sAPPα, in both CSF and brain. Overall, the effects of FAH65 in the rat study were greater than the clinically-tested BACE inhibitor verubecestat. The potential advantage of FAH65, which has BACE1 inhibition potency similar to that of Veru, is its high selectivity for inhibiting BACE1 cleavage of APP relative to other substrates such as NRG1 and PSGL1.

FAH65 has good drug-like properties, including stability in human plasma, microsomal stability, and water solubility. It also has a good hERG profile with an EC_50_ > 10 μM. The free unbound drug (*F*_*u*_) of 5.4 % for mouse brain tissue protein binding suggests the unbound brain concentration after 30mkd oral administartion of FAH65-E(-) at C_max_ is 9.6 nM, which is close to its IC_50_ for BACE1 inhibition (IC_50_ ∼ 5 nM) in the cell free enzyme assay but less than the predetermined *in vitro* efficacious dose in CHO-7W cells (EC_50_ = 25 nM, Fig. 6D). The oral administration of FAH65E(-) at this dose is likely to inhibit BACE1 less than 50 % in the brain.

We note ASBI therapy with FAH65 herein may only result in partial, not complete, inhibition of BACE1 in the brain. Such a partial BACE1 inhibition therapeutic approach is supported by the previous finding of McConlogue et al. [38] that indicated, at least in a mouse model of AD, that only a 12 % decrease in Aβ levels in brain results in dramatic reduction in plaque pathology. The potential of modest BACE1 inhibition and partial reduction of Aβ production in the brain was also suggested by Satir et al. [39], who found that less than a 50 % decrease in Aβ secretion by a BACE inhibitor – a low-level BACE1 inhibition magnitude [40] similar to that anticipated for FAH65E(-), or seen in the presence of the protective Icelandic mutation that results in ∼ 30 % lifelong reduction in BACE cleavage of APP [14] – does not impair synaptic function and thus is not likely to cause the cognitive deterioration observed with some BACE inhibitors that have failed in the clinic [41]

Despite reported target engagement, BACE inhibitors that have been studied in the clinic have not elicited improvements in cognitive performance, and in some instances have been associated with worsened cognitive performance [41]. The failure of BACE inhibitors in the clinic to-date has been attributed to off-target effects due to inhibition of non-APP BACE substrates, a limitation that an ASBI specifically addresses and is likely to overcome.

If successfully developed, a small molecule ASBI therapeutic with good oral bioavailability and brain penetrance may also likely have advantages over the currently approved amyloid-targeted therapeutic aducanumab and a similar monoclonal antibody-based therapy such as lecanemab [15,16] that only provided a modest decrease in decline of memory, in ease of delivery – oral dosing vs. infusion – and safety risks such as inflammation, ARIA, and risk of bleeding in the brain.

In our next steps toward preclinical development of this ASBI lead candidate, the active enantiomer of FAH65E(-), will undergo *in vivo* testing in additional murine models to assess effects on BACE1 activity/substrate cleavage, including off-target effects, in brain as well as other IND-enabling studies.

An ASBI that reaches the clinical testing stage may be most beneficial for patients before the onset of symptoms, for example, in persons who possess ApoE4 alleles or who are otherwise at risk for increased β-secretase processing of APP. FAH65E(-) may also provide the possibility of combination with FDA-approved mAb-based therapy with the potential to increase efficacy of both therapeutics by decreasing Aβ production while clearing existing amyloid; it may also allow a decrease in the dose of the mAb and a reduction in observed clinical side effects. Finally, FAH65E(-) could serve as a orally administered maintenance therapy to normalize Aβ levels after amyloid is cleared from the brain.

## Disclosures

The FAH series is part of the patent application UCLA-2018-200 and the issued claims in UCH-14160. Authors have nothing to disclose.

## Author contributions

JC performed in vitro assays and analyzed data; BJ participated in developing medical chemistry and analog design, OD performed the screening assay and in vitro studies, WC and JL performed pharmacokinetic and ADME analyses, DW performed characterization of FAH65 enantiomers, QZ developed the SEAP assays, OG and CZ performed/assisted with in vivo studies, KP, MJ, and CE performed assays, CPL performed the APP binding studies, JC and SP conducted the MSD analysis, PS performed in vivo studies, analyzed and graphed data and wrote the manuscript, DB and VJ originated the concept of ASBI identification and designed the screening assay, medicinal chemistry strategy and analog generation, in vitro and in vivo studies, analyzed data and edited the manuscript.

## Declaration of competing interest

The authors declare the following financial interests/personal relationships which may be considered as potential competing interests: Varghese John's Lab reports financial support was provided by NantNeuro LLC. Varghese John has patent pending to UCLA. The other authors declare that they have no known competing financial interests or personal relationships that could have appeared to influence the work reported in this paper.
